# Drug Repurposing of Verapamil for Radiation-Induced Enteropathy: Regulation of 5-LOX, Nrf2, and Intestinal Barrier Function

**DOI:** 10.3390/ph19091346

**Published:** 2026-08-26

**Authors:** Mehmet Fatih Dasiran, Hassen Daghmoura, Ahmet Akbaş, Yavuz Selim Angın, Bakiye Akbaş, Hatice Aygun, Yiğit Uyanıkgil, Oytun Erbas

**Affiliations:** 1 Department of General Surgery, Faculty of Medicine, Tokat Gaziosmanpasa University, 60230 Tokat, Türkiye; fatihdasiran@yahoo.com; 2 Department of General Surgery, Faculty of Medicine, Bezmialem Vakif University, 34093 Istanbul, Türkiye; hasgagh@hotmail.com; 3 Department of General Surgery, Faculty of Medicine, Karadeniz Technical University, 61080 Trabzon, Türkiye; 4 Department of General Surgery, Faculty of Medicine, Eskisehir Osmangazi University, 26040 Eskisehir, Türkiye; dryavuz05@gmail.com; 5 Department of Obstetrics and Gynecology, Faculty of Medicine, Karadeniz Technical University, 61080 Trabzon, Türkiye; bakiyeakbas@ktu.edu.tr; 6 Neuroscience Laboratory, BAMER, Biruni University, 34015 Istanbul, Türkiye; hatice_5aygun@hotmail.com; 7 Department of Histology and Embryology, Faculty of Medicine, Ege University, 35040 Izmir, Türkiye; yigit.uyanikgil@ege.edu.tr; 8 BAMER, Biruni University, 34015 Istanbul, Türkiye; oytunerbas2012@gmail.com

**Keywords:** radiotherapy, verapamil, radiation enteropathy, TNF-α, 5-LOX, Nrf-2, ZO-1

## Abstract

**Aim:** Radiation-induced intestinal injury is one of the major dose-limiting complications of abdominal radiotherapy and is closely associated with oxidative stress, inflammation, and disruption of epithelial barrier integrity. The present study aimed to investigate the protective effects of verapamil on radiation-induced small-intestinal injury in rats by evaluating histopathological alterations together with tissue levels of TNF-α, 5-LOX, Nrf2, and ZO-1 as markers related to inflammatory, redox-associated, and tight-junction-associated responses. **Materials and Methods:** Thirty female Wistar albino rats were divided into three groups: Normal Control (n = 10), Radiation + Saline (RAD, n = 10), and Radiation + Verapamil (n = 10). Whole-abdomen irradiation was performed using a single 12 Gy dose delivered by a 6 MV linear accelerator. Verapamil (10 mg/kg/day, s.c.) treatment was started 5 days before irradiation and continued for 5 days afterward. Histopathological injury in jejunal tissues was evaluated using hematoxylin–eosin staining. Tissue levels of TNF-α, 5-LOX, Nrf2, and ZO-1 were measured using ELISA. **Results:** Whole-abdomen irradiation caused marked histopathological injury characterized by villus disruption, glandular degeneration, and mucosal architectural damage. Irradiation significantly increased small-intestinal TNF-α and 5-LOX levels while decreasing Nrf2 and ZO-1 levels compared with the Normal Control group (*p* < 0.05). Verapamil treatment significantly attenuated radiation-induced histopathological injury and reduced TNF-α and 5-LOX levels. TNF-α returned to values comparable with the Normal Control group, whereas 5-LOX remained significantly elevated. Verapamil also partially restored Nrf2 and ZO-1 levels compared with the RAD group (*p* < 0.05), although both remained significantly lower than control levels. **Conclusions:** Verapamil attenuated radiation-induced small-intestinal injury and was associated with favorable modulation of TNF-α, 5-LOX, Nrf2, and ZO-1 levels. The protective response was partial, with persistent differences in 5-LOX, Nrf2, and ZO-1 compared with controls. These biomarker changes should not be interpreted as direct evidence of functional pathway activation or preservation of intestinal barrier function. Further studies incorporating direct mechanistic and functional assessments are warranted before clinical translation.

## 1. Introduction

Cancer remains one of the leading causes of morbidity and mortality worldwide, with GLOBOCAN 2022 estimating nearly 20 million new cases and 9.7 million deaths, and with the global burden expected to rise further in the coming decades [1]. Radiotherapy is therefore indispensable in modern oncology, and evidence-based utilization analyses indicate that approximately half of all patients with cancer will require radiotherapy at some point during the course of their disease [2,3]. Although the antitumor efficacy of radiotherapy is well established, its full therapeutic potential is still constrained by dose-limiting injury to normal tissues located within or adjacent to the irradiated field [4,5].

Among these normal tissues, the gastrointestinal tract is particularly sensitive to ionizing radiation, and the small intestine and colon are especially vulnerable because of their rapid epithelial turnover, high stem-cell activity within the crypt compartment, intense immune surveillance, and dependence on intact epithelial junctions for barrier homeostasis [5,6,7]. Clinically, this vulnerability may manifest during abdominal or pelvic radiotherapy as diarrhea, abdominal pain, urgency, nausea, rectal bleeding, and weight loss in the acute phase, and as fibrosis, strictures, fistula formation, malabsorption, and chronic bowel dysfunction in the late phase [3,4,5]. As a result, gastrointestinal toxicity remains a major factor limiting radiation dose intensification and may compromise treatment continuity, symptom control, and long-term quality of life in cancer patients.

The pathogenesis of radiation-induced intestinal injury is complex and multifactorial. Radiation-induced intestinal injury arises from direct DNA damage and water-radiolysis-driven oxidative stress that rapidly causes crypt/stem cell apoptosis, mitochondrial and microvascular dysfunction, and epithelial barrier disruption, which in turn activates DAMP-, TLR4-, and inflammasome-linked inflammatory pathways together with evolving dysbiosis to sustain cytokine production, bacterial translocation, permeability, and defective mucosal repair [8,9,10]. TNF-α is a key pro-inflammatory mediator in radiation-associated intestinal inflammation [8]. In parallel, 5-LOX-dependent eicosanoid pathways contribute to leukotriene-mediated inflammatory signaling in intestinal tissue [11,12]. In parallel, impairment of endogenous antioxidant defense systems, especially those governed by nuclear factor erythroid 2-related factor 2 (Nrf2), further aggravates oxidative tissue damage, whereas disruption of tight-junction proteins such as zonula occludens-1 (ZO-1) weakens epithelial cohesion and promotes barrier failure [13,14,15]. Accordingly, TNF-α, 5-LOX, Nrf2, and ZO-1 were selected as complementary tissue biomarkers representing distinct but biologically interconnected aspects of radiation-induced intestinal injury: cytokine-mediated inflammation, eicosanoid-related inflammatory signaling, redox-associated cytoprotective responses, and tight-junction-associated epithelial alterations, respectively. Their combined assessment was intended to provide a broader molecular context for the histopathological findings rather than to establish functional activation or inhibition of these pathways.

Verapamil is a clinically established non-dihydropyridine L-type calcium channel blocker that has long been used in the treatment of hypertension, angina, and supraventricular arrhythmias [16]. Its established clinical use and well-characterized pharmacological profile also make verapamil relevant from a drug-repurposing perspective. Drug repurposing evaluates an existing medicine for a new therapeutic indication and can facilitate more rapid clinical development while reducing some of the uncertainty associated with de novo drug development [17]. However, interest in verapamil has progressively expanded beyond cardiovascular pharmacology because this drug appears to exert biologic effects that intersect with oxidative, inflammatory, and cell-survival pathways [18,19]. In non-intestinal experimental systems, verapamil has been shown to activate Nrf2 through p62-dependent autophagic degradation of Keap1, thereby enhancing cytoprotective antioxidant responses [18]. In addition, verapamil has been shown to attenuate oxidative stress and tissue injury in non-intestinal models, including myocardial ischemia–reperfusion damage, supporting the concept that calcium-channel modulation may influence redox-sensitive inflammatory injury pathways more broadly [19]. These pleiotropic properties make verapamil an attractive repurposing candidate for radiation-induced intestinal injury, where calcium dysregulation, oxidative stress, and inflammation converge. Taken together, these observations provide biological plausibility for evaluating verapamil as a repurposing candidate in radiation-induced intestinal injury, without assuming that the same molecular mechanisms necessarily operate in irradiated intestinal tissue.

Evidence directly relevant to the gastrointestinal tract, although still limited, is encouraging. In experimental colitis, verapamil was shown to alter mucosal eicosanoid synthesis and accelerate healing, suggesting that it can influence leukotriene-associated inflammatory damage in gut tissue [20]. In a rat intestinal ischemia–reperfusion model, verapamil also reduced tissue injury, indicating that its protective effects may extend to intestinal conditions driven by oxidative and inflammatory stress [21]. When these bowel-specific observations are considered together with studies showing verapamil-mediated activation of Nrf2 and attenuation of oxidative injury in other organ systems, the drug emerges as a biologically plausible radioprotective strategy for intestinal tissue that warrants direct evaluation [18,19]. However, whether these observations extend to radiation-induced small-intestinal injury, particularly in relation to concurrent changes in TNF-α, 5-LOX, Nrf2, and ZO-1, remains insufficiently characterized.

Despite this rationale, the effect of verapamil on radiation-induced small-intestinal injury remains insufficiently defined. Therefore, the present study was designed to evaluate the effects of verapamil on whole-abdomen irradiation-induced small-intestinal injury in rats by assessing histopathological changes together with tissue levels of TNF-α, 5-LOX, Nrf2, and ZO-1 as complementary markers of inflammatory, redox-associated, and tight-junction-related responses.

## 2. Results

### 2.1. Histopathological Score

Histopathological injury scores of the small intestine did not demonstrate normal distribution according to the Shapiro–Wilk test; therefore, non-parametric analyses were performed. Histopathological scores were expressed as median [interquartile range (IQR)] together with mean ± SD values for descriptive purposes.

A significant overall difference was observed among the groups in terms of histopathological injury scores (Kruskal–Wallis χ^2^ = 19.782, df = 2, *p* < 0.001). The Control group demonstrated minimal histopathological alterations (median: 0.0 [0.0–1.0]; mean ± SD: 0.30 ± 0.48), whereas the RAD + Saline group exhibited markedly increased intestinal injury scores (median: 3.0 [2.5–4.0]; mean ± SD: 3.22 ± 0.83). In the RAD + Verapamil group, histopathological injury scores were significantly reduced compared with the RAD + Saline group (median: 1.0 [0.75–2.0]; mean ± SD: 1.30 ± 0.95).

Pairwise comparisons using Dunn’s multiple-comparison test demonstrated that histopathological injury scores were significantly higher in the RAD + Saline group than in the Control group (*p* < 0.0001). Verapamil treatment significantly reduced histopathological injury scores compared with the RAD + Saline group (*p* = 0.0254), whereas no significant difference was observed between the RAD + Verapamil and Control groups (*p* = 0.1937).

**Figure 1:**
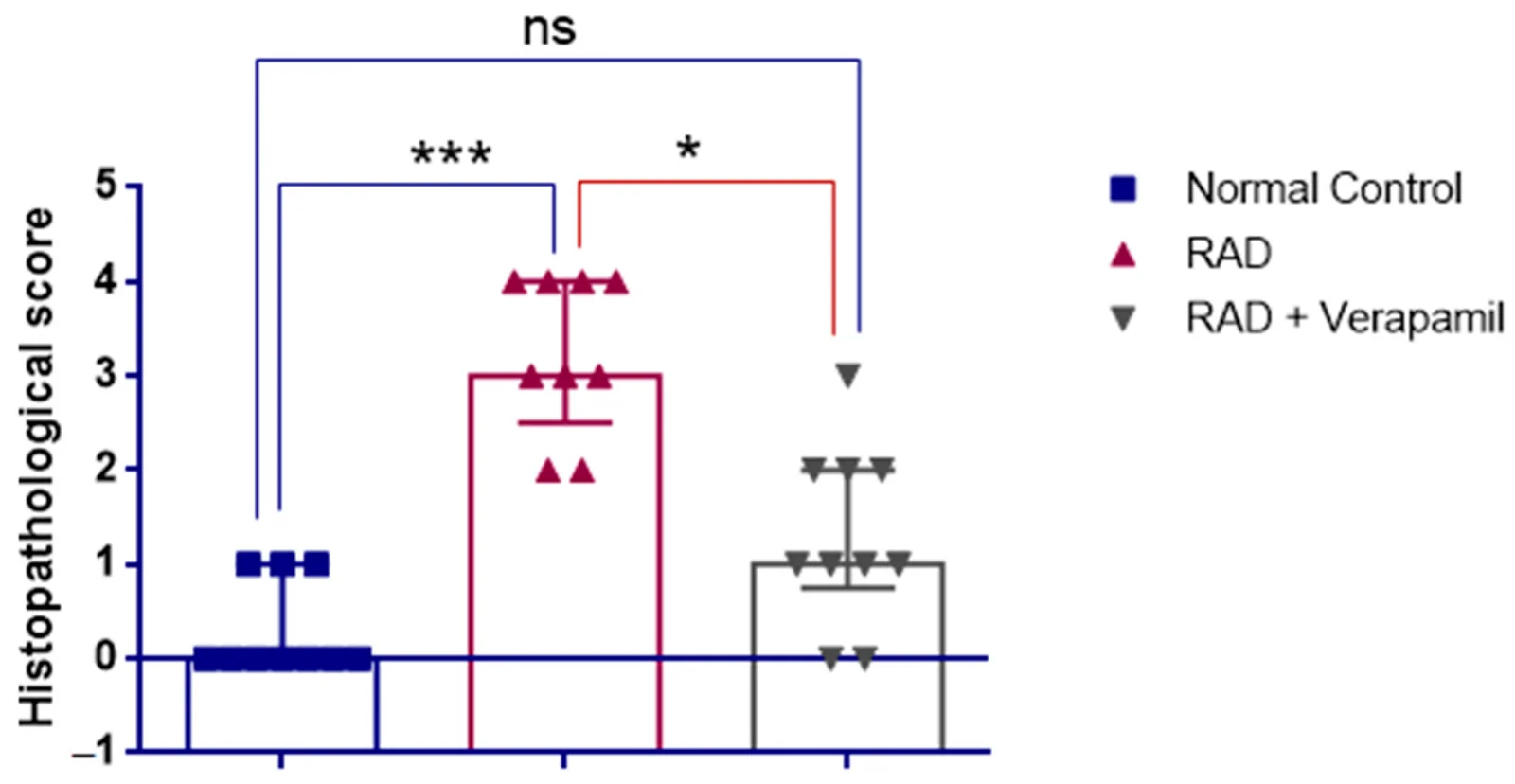
Histopathological injury scores of small intestine tissues in the experimental groups. Whole-abdomen irradiation significantly increased intestinal histopathological damage in the RAD group compared with the Normal Control group, whereas verapamil treatment significantly reduced radiation-induced tissue injury. No significant difference was observed between the Normal Control and RAD + Verapamil groups. Data are presented as median [IQR]. * *p* < 0.05, *** *p* < 0.001; ns, not significant. Normal Control: n = 10; RAD: n = 9; RAD + Verapamil: n = 10.

**Figure 2:**
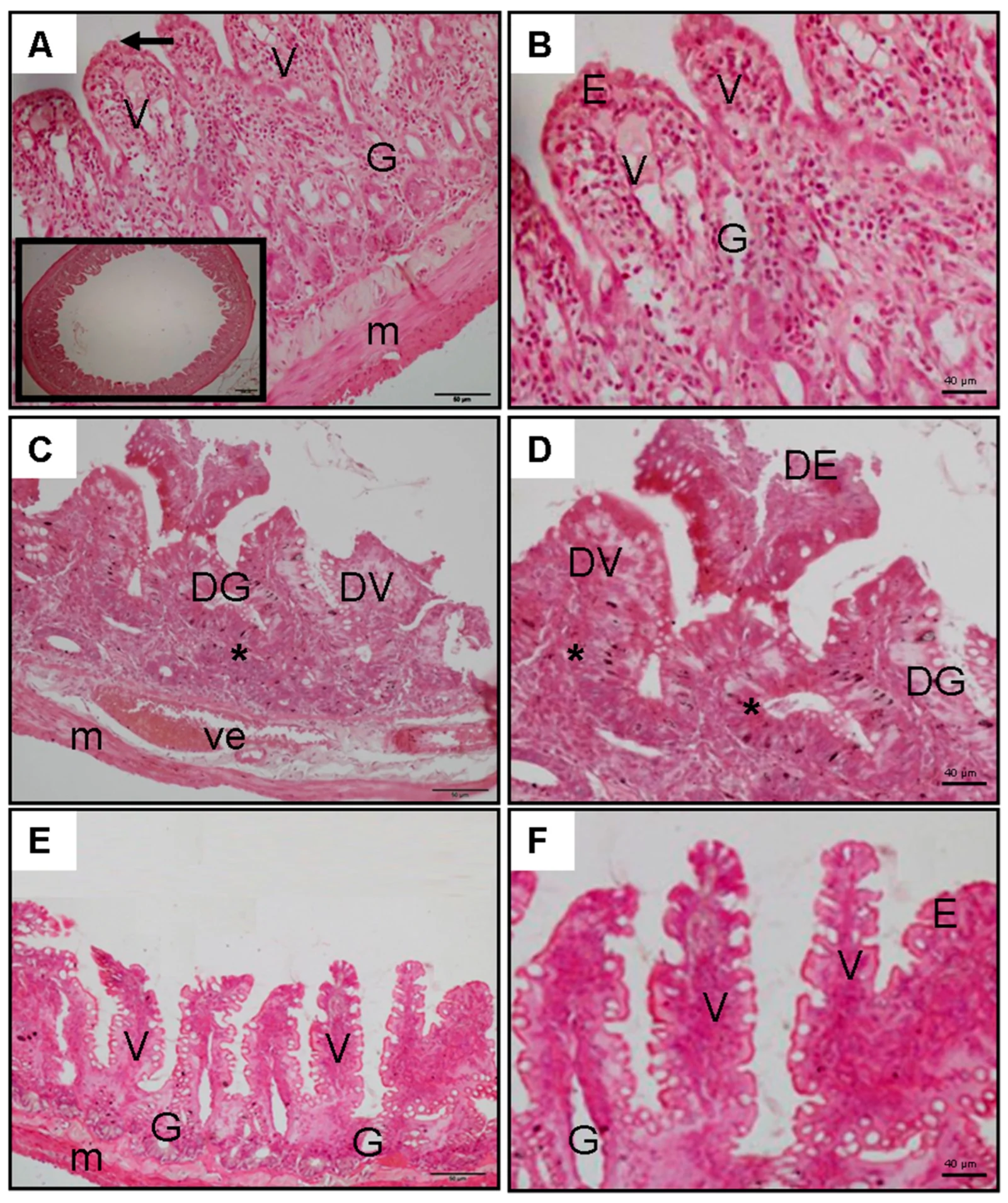
Representative histopathological images of small intestine tissues stained with hematoxylin and eosin (H&E) (original magnifications ×20 and ×40). (**A**,**B**) Normal Control group: Small intestine sections demonstrated preserved intestinal architecture with intact epithelium (arrow and E), regularly organized villi (V), normal Lieberkühn glands/crypts (G), and intact muscular layer (m). No evidence of epithelial disruption, glandular degeneration, or inflammatory tissue injury was observed. (**C**,**D**) Radiation + 0.9% NaCl group: Irradiated small intestine tissues exhibited marked histopathological injury characterized by disrupted villi (DV), degeneration of Lieberkühn glands/crypts (DG), epithelial destruction (DE), loss of normal mucosal organization with disrupted tissue architecture (asterisk), inflammatory cellular infiltration, and dilated small veins (ve), intact muscular layer (m). These findings indicate severe radiation-induced mucosal damage. (**E**,**F**) Radiation + Verapamil group: Verapamil-treated irradiated tissues showed partial preservation of intestinal morphology, including relatively preserved villi (V), glandular structures (G), and epithelial integrity (E), intact muscular layer (m). Histopathological injury and mucosal disruption were partially attenuated compared with the Radiation + 0.9% NaCl group, supporting a protective effect of verapamil against radiation-induced intestinal damage.

These findings indicate that whole-abdomen irradiation induced marked histopathological damage in the small intestine, whereas verapamil treatment significantly attenuated radiation-associated intestinal injury (Figure 1 and Figure 2).

### 2.2. Small Intestine TNF-α Level

Before comparative analyses, normality and variance homogeneity assumptions were evaluated. Shapiro–Wilk analysis demonstrated that TNF-α values were normally distributed in all groups (*p* > 0.05). In addition, variance homogeneity assumptions were satisfied according to both the Brown–Forsythe test (F(2,26) = 0.9315, *p* = 0.4067) and Bartlett’s test (*p* = 0.4091). Therefore, parametric analyses were performed.

One-way ANOVA revealed a statistically significant difference among the groups in small intestine TNF-α levels (F(2,26) = 50.28, *p* < 0.0001, R^2^ = 0.7946). The RAD group demonstrated significantly elevated TNF-α levels (290.6 ± 29.35 pg/mg) compared with the Normal Control group (186.8 ± 18.24 pg/mg, *p* < 0.0001). In contrast, verapamil treatment significantly reduced TNF-α levels in irradiated rats (201.8 ± 24.25 pg/mg) compared with the RAD group (*p* < 0.0001). No statistically significant difference was observed between the Normal Control and RAD + Verapamil groups (*p* = 0.3624).

**Figure 3:**
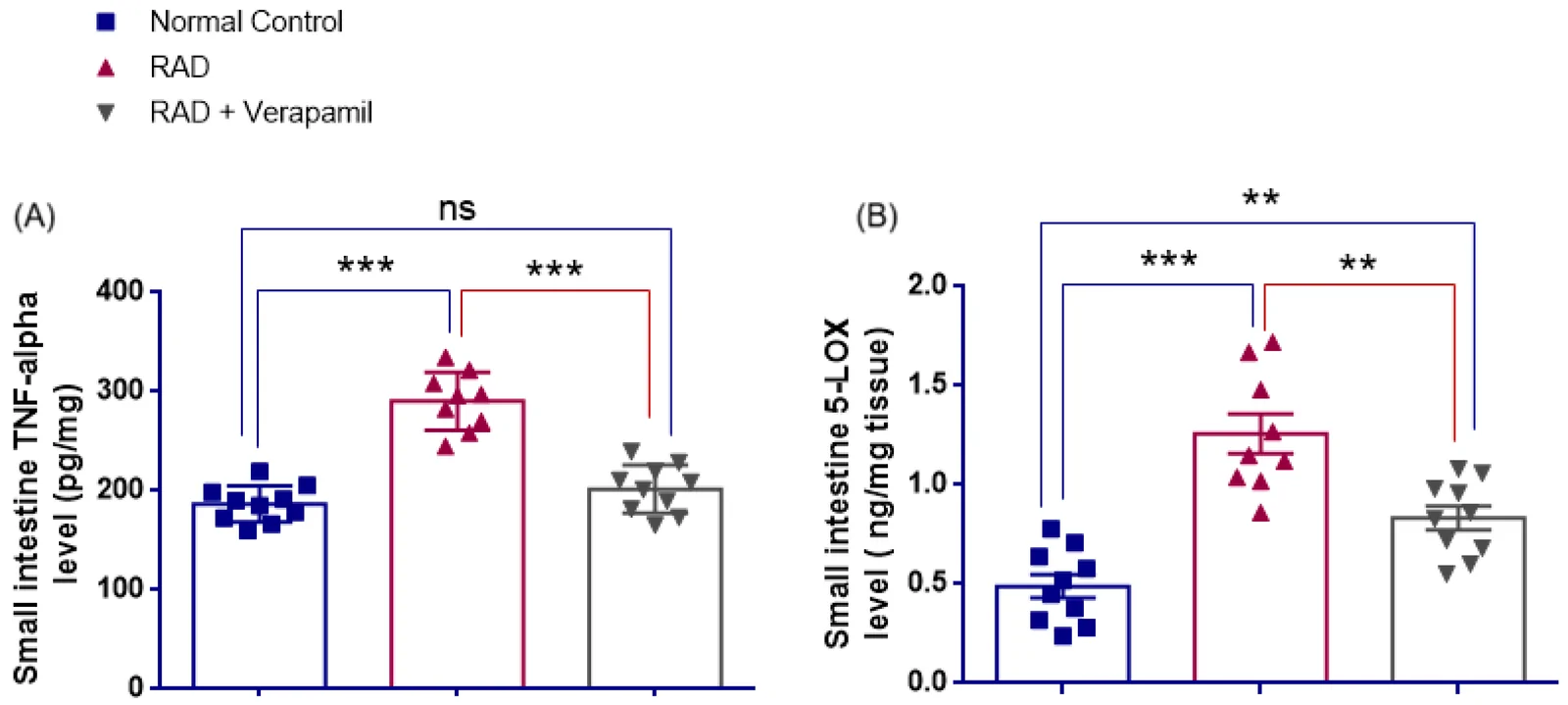
Effects of whole-abdomen irradiation and verapamil treatment on small intestine TNF-α and 5-LOX levels. (**A**) Small intestine TNF-α levels and (**B**) small intestine 5-LOX levels were significantly increased in the RAD group compared with the Normal Control group. Verapamil treatment significantly reduced both TNF-α and 5-LOX levels in irradiated rats. No significant difference in TNF-α levels was observed between the Normal Control and RAD + Verapamil groups, whereas 5-LOX levels remained significantly higher in the RAD + Verapamil group compared with the Normal Control group. Data are presented as mean ± SD. ** *p* < 0.01, *** *p* < 0.001; ns, not significant. Normal Control: n = 10; RAD: n = 9; RAD + Verapamil: n = 10.

These findings indicate that whole-abdomen irradiation markedly increased intestinal TNF-α levels, whereas verapamil treatment was associated with a substantial reduction in radiation-induced TNF-α elevation (Figure 3A).

### 2.3. Small Intestine 5-LOX Level

Shapiro–Wilk analysis demonstrated that 5-LOX values were normally distributed in all groups (*p* > 0.05). In addition, variance homogeneity assumptions were satisfied according to both the Brown–Forsythe test (F(2,26) = 0.9797, *p* = 0.3889) and Bartlett’s test (*p* = 0.2764). Therefore, parametric analyses were performed.

One-way ANOVA demonstrated a statistically significant difference among the groups in small intestine 5-LOX levels (F(2,26) = 26.79, *p* < 0.0001, R^2^ = 0.6733). The RAD group showed significantly elevated 5-LOX levels (1.259 ± 0.301 ng/mg tissue) compared with the Normal Control group (0.490 ± 0.187 ng/mg tissue, *p* < 0.0001). Verapamil treatment significantly reduced 5-LOX levels in irradiated rats (0.832 ± 0.189 ng/mg tissue) compared with the RAD group (*p* = 0.0011). However, 5-LOX levels in the RAD + Verapamil group remained significantly higher than those in the Normal Control group (*p* = 0.0069) (Figure 3B).

### 2.4. Small Intestine Nrf-2 Level

**Figure 4:**
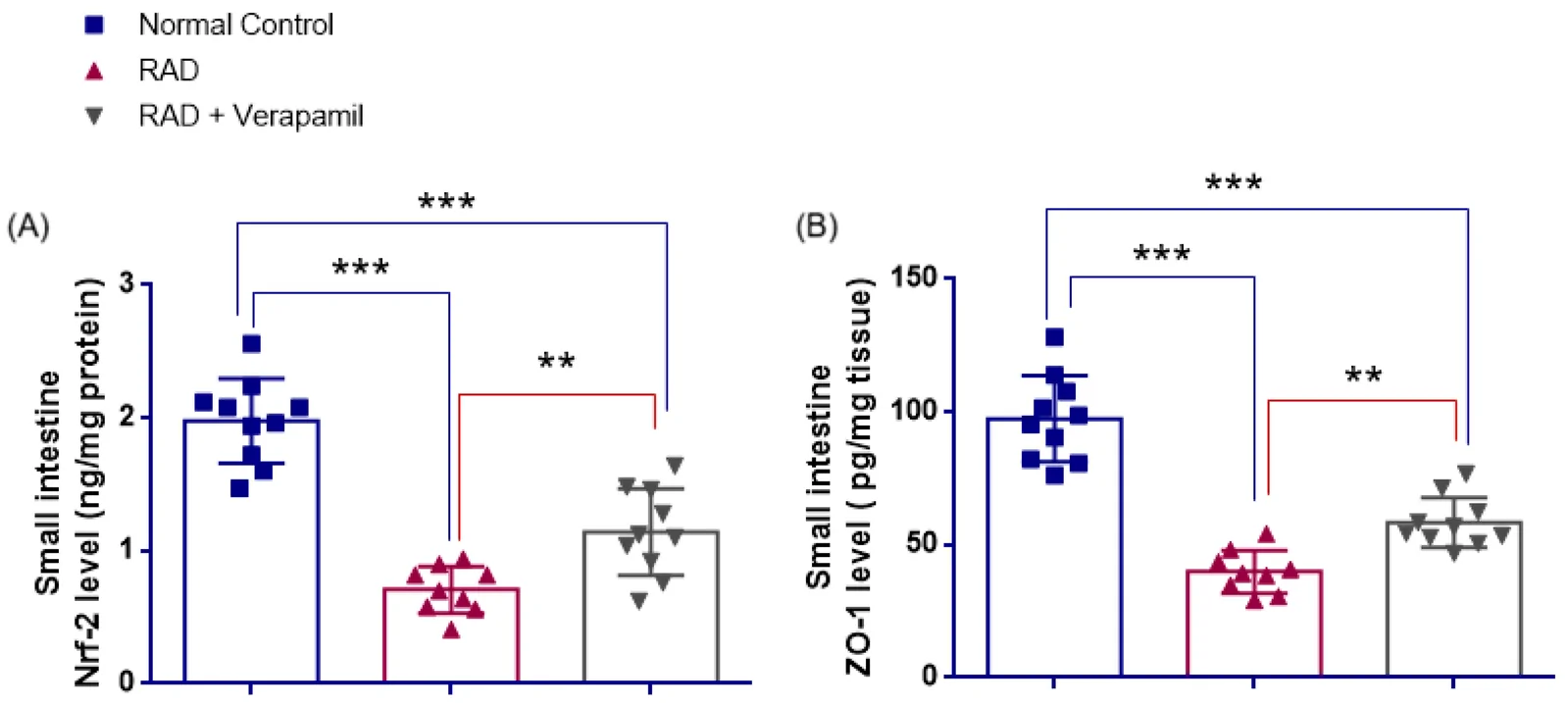
Effects of whole-abdomen irradiation and verapamil treatment on small intestine Nrf-2 and ZO-1 levels. (**A**) Small intestine Nrf2 levels and (**B**) small intestine ZO-1 levels were significantly decreased in the RAD group compared with the Normal Control group. Verapamil treatment significantly increased both Nrf2 and ZO-1 levels in irradiated rats; however, these parameters remained significantly lower than those of the Normal Control group. Data are presented as mean ± SD. ** *p* < 0.01, *** *p* < 0.001. Normal Control: n = 10; RAD: n = 9; RAD + Verapamil: n = 10.

Figure 4A shows that small intestine Nrf-2 levels were significantly altered among the experimental groups. Shapiro–Wilk analysis demonstrated that Nrf-2 values were normally distributed in all groups (*p* > 0.05). In addition, variance homogeneity assumptions were satisfied according to both the Brown–Forsythe test (F(2,26) = 1.155, *p* = 0.3306) and Bartlett’s test (*p* = 0.1992). Therefore, parametric analyses were performed.

One-way ANOVA demonstrated a statistically significant difference among the groups regarding small intestine Nrf-2 levels (F(2,26) = 48.81, *p* < 0.0001, R^2^ = 0.7897). The RAD group demonstrated significantly reduced Nrf-2 levels (0.708 ± 0.176 ng/mg protein) compared with the Normal Control group (1.977 ± 0.319 ng/mg protein, *p* < 0.0001). Verapamil treatment significantly increased Nrf-2 levels in irradiated rats (1.142 ± 0.327 ng/mg protein) compared with the RAD group (*p* = 0.0076). However, Nrf-2 levels in the RAD + Verapamil group remained significantly lower than those in the Normal Control group (*p* < 0.0001).

These findings indicate that whole-abdomen irradiation markedly reduced intestinal Nrf-2 levels, whereas verapamil treatment was associated with a significant but partial recovery of Nrf-2 levels in small intestine tissue.

### 2.5. Small Intestine ZO-1 Level

As presented in Figure 4B, small intestine ZO-1 levels differed significantly among the study groups. Assessment of data distribution with the Shapiro–Wilk test indicated normal distribution in all groups (*p* > 0.05). Homogeneity of variances was also confirmed by both the Brown–Forsythe analysis (F(2,26) = 2.303, *p* = 0.1200) and Bartlett’s test (*p* = 0.0958). Based on these findings, parametric statistical methods were considered appropriate for subsequent analyses.

One-way ANOVA demonstrated a statistically significant difference among the groups in small intestine ZO-1 levels (F(2,26) = 58.98, *p* < 0.0001, R^2^ = 0.8194). The RAD group showed significantly reduced ZO-1 levels (39.90 ± 8.06 pg/mg tissue) compared with the Normal Control group (97.48 ± 16.19 pg/mg tissue, *p* < 0.0001). Verapamil treatment significantly increased ZO-1 levels in irradiated rats (58.33 ± 9.34 pg/mg tissue) compared with the RAD group (*p* = 0.0063). However, ZO-1 levels in the RAD + Verapamil group remained significantly lower than those in the Normal Control group (*p* < 0.0001).

These findings indicate that whole-abdomen irradiation markedly reduced intestinal ZO-1 levels, whereas verapamil treatment was associated with a significant but partial recovery of ZO-1 levels in small intestine tissue.

## 3. Discussion

In the present study, whole-abdomen irradiation produced a reproducible pattern of small-intestinal injury characterized by histopathological disruption, increased TNF-α and 5-LOX levels, reduced Nrf2 and ZO-1 levels, and partial but significant recovery after verapamil treatment. Collectively, these findings demonstrate parallel changes in biomarkers related to inflammatory, redox-associated, and tight-junction-associated responses accompanying radiation-induced intestinal injury, rather than establishing functional alterations in these pathways.

In our study, whole-abdomen irradiation induced a typical acute radiation enteropathy phenotype in the jejunum, including villus shortening, epithelial denudation, crypt/gland degeneration, vascular dilatation, and disruption of mucosal organization, whereas verapamil markedly attenuated these alterations, supporting a genuine tissue-protective effect in the irradiated intestine [7,8,22]. These findings are consistent with previous experimental studies showing that irradiation disrupts crypt–villus architecture, impairs epithelial renewal, and promotes inflammatory and microvascular injury in the intestinal mucosa [23,24,25]. Because crypt epithelial injury is closely associated with gastrointestinal radiation syndrome severity and delayed mucosal recovery, the relative preservation of villi, glands, and epithelial continuity in the verapamil-treated group is consistent with attenuation of structural mucosal injury, although regenerative capacity was not directly assessed [5,22,26]. This interpretation is further supported by our ZO-1 findings, which showed partial recovery of this tight-junction-associated protein after verapamil treatment; however, functional barrier integrity was not directly assessed [14,27,28]. Taken together, the close agreement between our histopathological and biomarker findings supports the validity of our model and suggests that verapamil may limit radiation-induced mucosal injury, with accompanying changes in ZO-1 levels that are consistent with, but do not directly demonstrate, preservation of barrier function.

The inflammatory data further support this interpretation. In our study, irradiation markedly increased intestinal TNF-α and 5-LOX levels, whereas verapamil significantly reduced both protein levels, consistent with attenuation of radiation-associated inflammatory changes [8,20]. This pattern is mechanistically plausible because TNF-α is a central mediator of radiation enteropathy that amplifies leukocyte recruitment, epithelial apoptosis, endothelial activation, and persistence of mucosal inflammation after irradiation [8,15]. The 5-LOX finding adds an important second layer to this interpretation because 5-LOX converts arachidonic acid into leukotrienes that sustain inflammatory-cell trafficking, microvascular dysfunction, and tissue injury in the intestine [29,30]. These pathways are unlikely to act independently, since TNF-rich inflammatory states favor arachidonic acid mobilization and downstream eicosanoid signaling, while leukotriene-rich signaling further amplifies cytokine-driven mucosal damage [29,30]. Such interactions may also be relevant to barrier-related injury, as TNF-α and radiation-associated oxidative stress disrupt tight junction organization and worsen epithelial permeability in intestinal models [9,13,14,31]. The concomitant reduction in Nrf2 in our irradiated animals, together with its partial recovery after verapamil, is also consistent with the involvement of redox-associated responses in this inflammatory context [15]. Accordingly, the parallel decrease in TNF-α and 5-LOX after verapamil is consistent with modulation of cytokine- and eicosanoid-related inflammatory responses; however, 5-LOX enzymatic activity and leukotriene production were not directly measured, and causal pathway modulation cannot be established from the present data [20,29].

Changes in Nrf2 provide the redox-associated counterpart of this inflammatory picture. In our model, irradiation markedly reduced small-intestinal Nrf2 levels, whereas verapamil significantly increased Nrf2 levels, although recovery remained incomplete. This finding fits well with contemporary understanding of Nrf2 as a master transcriptional regulator of antioxidant defense and cytoprotection under radiation stress. Recent animal work has consistently linked preserved or activated Nrf2 signaling with reduced radiation-induced intestinal injury. Zhu et al. [32] showed that quercetin mitigated radiation-induced intestinal injury and promoted regeneration through an Nrf2-mediated antioxidant pathway, while Zhang et al. [33] demonstrated that ferulic acid protected the intestine through coordinated DJ-1/Nrf2 and Sirt1/NF-κB/NLRP3 signaling. Most importantly, Xu et al. [15] recently reported that loss of Nrf2 aggravates ionizing radiation-induced intestinal injury through activation of the cGAS/STING pathway via Pirin, which is consistent with the overall pattern observed in our study, in which lower Nrf2 levels accompanied more pronounced radiation-induced intestinal injury and verapamil treatment partially restored Nrf2 levels alongside attenuation of tissue damage. Although some earlier reports suggested context-dependent or paradoxical Nrf2 responses after irradiation [34], the overall direction of our findings aligns more closely with the current literature supporting a protective role of Nrf2 in radiation-induced intestinal injury. Against this background, the partial recovery of Nrf2 levels in the verapamil-treated group is consistent with involvement of Nrf2-related redox responses in the observed intestinal protection; however, Nrf2 pathway activation was not directly assessed, and the present findings do not establish a causal mechanism. Accordingly, the observed changes in Nrf2 and 5-LOX should be interpreted as associative biomarker responses; establishing causality will require pathway-specific experiments, such as pharmacological inhibition or genetic manipulation of Nrf2 and/or 5-LOX, together with direct assessment of Nrf2 transcriptional activity, downstream antioxidant targets, 5-LOX enzymatic activity, and leukotriene production.

A notable finding of the present study was the marked reduction in small-intestinal ZO-1 after irradiation and its significant, although incomplete, recovery with verapamil. This result is biologically relevant because radiation-induced intestinal injury is increasingly recognized as a barrier disorder as well as an epithelial injury state. Experimental studies show that irradiation rapidly disorganizes apical junctional complexes, downregulates ZO-1 and related tight-junction proteins, and increases epithelial permeability in mouse colon, irradiated intestinal tissue, and Caco-2 monolayers [9,14,31,35]. Because ZO-1 functions as a structural scaffold that stabilizes the tight-junction barrier, its reduction in our model is consistent with a tight-junction-associated alteration and potential barrier vulnerability; however, ZO-1 levels alone do not establish functional barrier impairment [36]. The parallel increase in TNF-α and 5-LOX provides additional biological context for this interpretation since TNF-α disrupts intestinal tight-junction organization, and leukotriene-linked pathways contribute to radiation-associated intestinal inflammation and microvascular dysfunction [13,30]. The concurrent reduction in Nrf2 is likewise biologically relevant, as Nrf2 deficiency aggravates irradiation-induced intestinal injury, and the direction of the verapamil response is compatible with prior reports linking verapamil to cytoprotective Nrf2 activation and altered intestinal eicosanoid signaling in non-radiation models [15,18,20]. Nevertheless, because we did not perform functional permeability assays, assess tight-junction protein localization, or directly evaluate Nrf2 pathway activation, these findings should be interpreted as associative and biologically supportive rather than as evidence of preserved barrier function or causal pathway modulation.

The 10 mg/kg/day verapamil dose was selected based on previous experimental studies demonstrating biological activity at this dose in models of oxidative and tissue injury [37,38], while Batçıoğlu et al. [39] specifically reported attenuation of irradiation-associated lipid peroxidation with verapamil. However, these studies involved different target tissues, administration routes, and experimental conditions and therefore do not establish 10 mg/kg/day as an optimal intestinal radioprotective dose. Because only a single dose was evaluated in the present study, future dose-ranging studies are required to define the minimum effective dose, dose–response relationship, and therapeutic window.

Only female rats were included in the present study to evaluate the protective effects of verapamil within a single biological context, with the single-sex design transparently reported and scientifically justified in accordance with current recommendations emphasizing consideration of sex as a biological variable [40,41,42]. The long-standing assumption that female animals are inherently more variable because of the estrous cycle has not been consistently supported by experimental evidence, and biological variability is not systematically greater in females than in males [43,44]. Moreover, experimental studies in diverse disease models have demonstrated that biological sex may influence molecular, immune, and inflammatory mechanisms rather than merely acting as a source of experimental variability [45,46,47]. In radiation research, sex-related differences in radiation-associated responses have been reported to vary across organ-specific outcomes rather than following a uniform pattern [48]. These observations collectively support the importance of considering biological sex as a relevant biological variable in experimental radiation research. Nevertheless, because sex-related differences are not consistently observed across all radiation-induced outcomes, the use of female rats in the present study should be viewed as a scientifically justified design choice rather than as implying that one sex is inherently more suitable than the other. Accordingly, because the present study was not designed to evaluate sex-dependent differences, the findings should be interpreted as female-specific, and future studies directly comparing both sexes are warranted to determine whether the protective effects of verapamil are influenced by biological sex [49,50].

From a translational perspective, the established clinical availability of verapamil may facilitate drug repurposing; however, the 10 mg/kg/day subcutaneous regimen used in the present rat model should not be directly extrapolated to patients, because clinically relevant exposure depends on formulation, route, pharmacokinetics, treatment duration, and tolerability. Oral verapamil has been administered for up to 12 months in clinical repurposing studies, including doses up to 360 mg/day, indicating that sustained exposure can be feasible in carefully selected populations [51,52,53,54]. Nevertheless, these non-oncology cohorts do not establish long-term safety in patients receiving abdominal or pelvic radiotherapy. With prolonged administration, clinically relevant cardiovascular concerns include symptomatic hypotension, sinus bradycardia, PR-interval prolongation, and higher-grade atrioventricular block, as well as worsening ventricular function related to verapamil’s negative inotropic effect, particularly in patients with pre-existing left ventricular dysfunction or concomitant beta-blocker or other cardiodepressant therapy [55]. Constipation is a common adverse effect, and rare non-obstructive ileus has also been reported; these effects could complicate gastrointestinal symptom assessment in patients already vulnerable to radiation-associated bowel dysfunction [3,4,55].

Drug-interaction screening is equally important in oncology. Verapamil may increase doxorubicin concentrations [55], R-verapamil has been reported to reduce paclitaxel clearance [56], and verapamil may substantially increase everolimus exposure [57]. CYP3A4 inhibitors may raise verapamil concentrations and increase the risk of hypotension or bradyarrhythmias [55]. Accordingly, future clinical studies should exclude patients where verapamil is contraindicated or otherwise carefully risk-stratify those with clinically significant hypotension, baseline bradyarrhythmia, sick sinus syndrome, second- or third-degree atrioventricular block without a functioning pacemaker, severe left ventricular systolic dysfunction, decompensated heart failure, clinically important bowel dysmotility, hepatic impairment, or unavoidable interacting medications [55]. Baseline medication reconciliation, blood pressure, resting heart rate, and electrocardiography should be obtained before treatment; assessment of ventricular function and liver tests should be added when clinically indicated [54,55]. During prolonged therapy, serial blood pressure and heart rate measurements, repeat electrocardiography after dose escalation or at the target dose, and active monitoring for constipation, ileus, volume depletion, conduction abnormalities, and heart failure symptoms would be appropriate [54,55]. Importantly, because verapamil was initiated 5 days before irradiation and continued for 5 days afterward in the present study, our findings primarily support a prophylactic/peri-irradiation approach and do not establish efficacy for treatment initiated after radiation injury. Future studies should therefore define clinically relevant dose–exposure relationships and treatment duration, directly compare prophylactic and therapeutic schedules, and evaluate long-term cardiovascular and gastrointestinal tolerability and compatibility with concomitant anticancer therapies before translation to patients undergoing radiotherapy.

## 4. Materials and Methods

### 4.1. Animals

Thirty female Wistar albino rats (10–12 weeks old, weighing 150–200 g) were obtained from the Experimental Animal Laboratory Center of Science University. Rats were maintained at 22 ± 2 °C under a 12 h light–dark cycle with free access to standard pellet chow and water ad libitum. The animals were housed in pairs in stainless-steel cages and were allowed to adapt to the laboratory environment for at least 1 week before the initiation of the experimental procedures.

All experimental procedures were carried out in accordance with the Guide for the Care and Use of Laboratory Animals published by the National Institutes of Health (NIH, Bethesda, MD, USA). The experimental protocol was approved by the Animal Ethics Committee of Science University (Approval No.: 22370204; Date: 4 January 2023).

### 4.2. Clinical Monitoring and Animal Welfare

Throughout the experimental period, all animals were monitored daily under the supervision of the veterinarian and animal health technician of the Experimental Animal Center. Body weight was recorded once daily, and food and water consumption were monitored throughout the study. Clinical observations included assessment of general health status, food and water intake, body weight changes, and the condition of the irradiated region. To facilitate feeding during the acute post-irradiation period, the standard pellet diet was lightly moistened with drinking water from the second day after irradiation. Humane endpoints were predefined according to the approved animal ethics protocol and included body weight loss exceeding 15%, persistent reduction in food or water intake, or any condition requiring veterinary intervention. One animal in the irradiated saline-treated group died because of irradiation-related complications before reaching the planned study endpoint. The remaining animals completed the experimental protocol without meeting the predefined humane endpoint criteria.

### 4.3. Experimental Protocol

**Figure 5:**
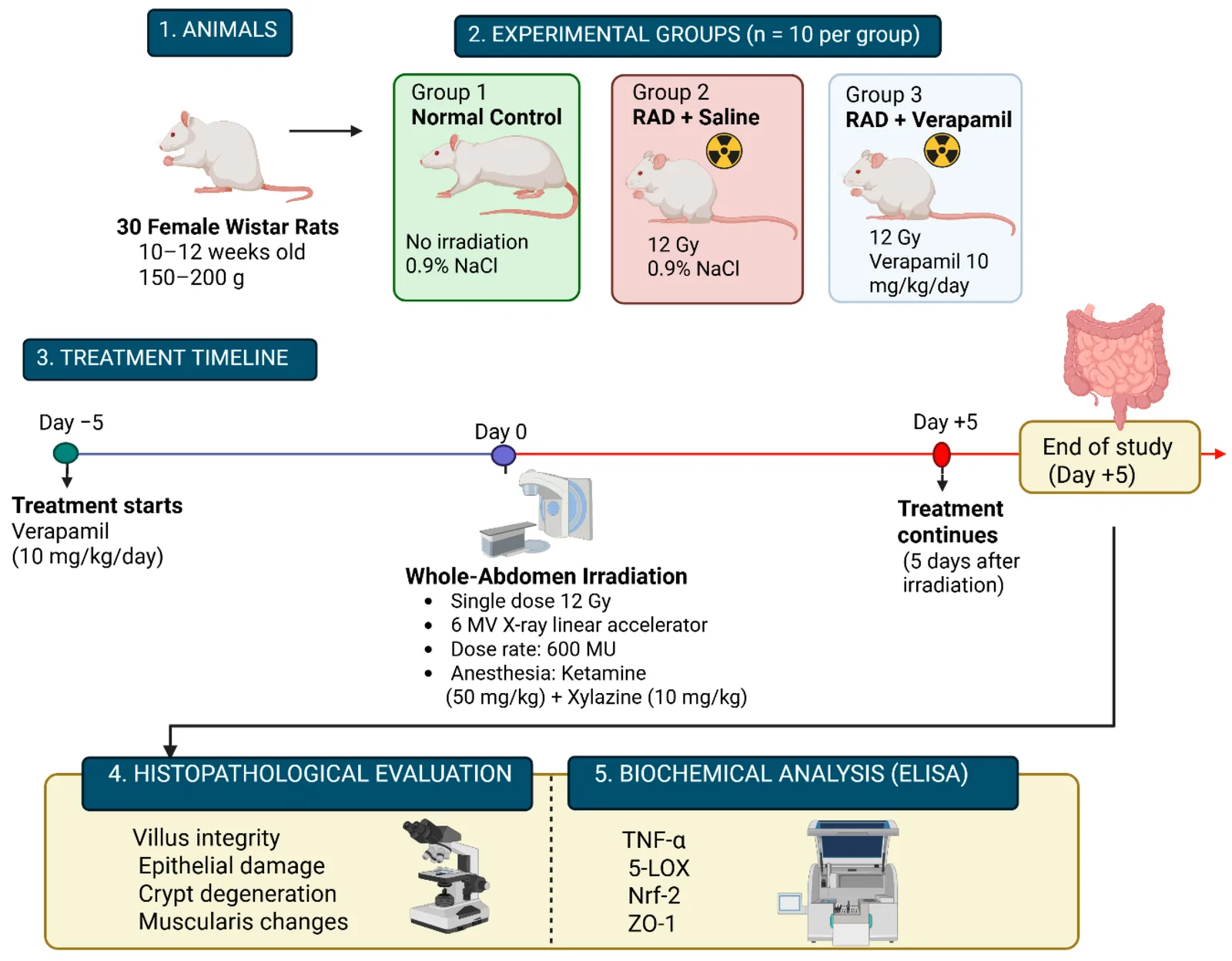
Schematic illustration of the experimental design and study workflow. Thirty female Wistar rats were randomly allocated into three groups: Normal Control, Radiation + Saline (RAD), and Radiation + Verapamil. Whole-abdomen irradiation was administered as a single 12 Gy dose using a 6 MV linear accelerator. Verapamil treatment (10 mg/kg/day, s.c.) was initiated 5 days before irradiation and continued for 5 days after irradiation. At the end of the experimental period, jejunum tissues were collected for histopathological evaluation and biochemical analyses. Histopathological assessment included evaluation of villus integrity, epithelial damage, crypt degeneration, and muscularis alterations. Tissue levels of TNF-α, 5-LOX, Nrf-2, and ZO-1 were determined by ELISA.

A total of 30 female Wistar albino rats were included in the study. Following the acclimatization period, animals were randomly assigned to the experimental groups using a computer-generated randomization sequence. Randomization was performed by the veterinarian and animal health technician of the Experimental Animal Center, who were independent of the experimental procedures, sample collection, histopathological evaluation, and biochemical analyses. To establish the radiation-induced intestinal injury model, whole-abdomen irradiation (RAD) was performed in 20 rats. Drug administrations were initiated 5 days before irradiation and continued for 5 days thereafter. The animals were allocated to the following experimental groups (Figure 5):

- Group 1: Normal Control group (n = 10)
  Rats received neither irradiation nor drug treatment and were administered 0.9% saline (1 mL/kg/day, s.c.) throughout the experimental period.
- Group 2: RAD + Saline group (n = 10)
  Rats underwent whole-abdomen irradiation and received 0.9% saline (1 mL/kg/day, s.c.) for 10 consecutive days.
- Group 3: RAD + Verapamil group (n = 10)
  Rats underwent whole-abdomen irradiation and were treated with verapamil (10 mg/kg/day, s.c.) for 10 consecutive days.

Drug treatments were started 5 days before irradiation and continued for 5 days following irradiation. During the experimental period, one rat in the RAD + Saline group died following irradiation-related complications and was excluded from the final biochemical and histopathological analyses. Therefore, the final evaluation was performed using nine rats in the RAD + Saline group and ten rats in the remaining groups.

At the end of the experimental period, all surviving animals were deeply anesthetized with ketamine/xylazine. Following anesthesia, blood samples were collected by cardiac puncture for biochemical analyses. Subsequently, the animals were euthanized by cervical dislocation, and small intestine (jejunum) tissues were harvested for histopathological and biochemical examinations (Figure 5).

### 4.4. Radiotherapy

Whole-abdomen irradiation procedures were performed under ketamine (50 mg/kg) and xylazine (10 mg/kg) anesthesia to minimize animal discomfort during the procedure. After adequate anesthesia was achieved, the rats were positioned in the supine position on a Plexiglas platform, and their extremities were gently secured to ensure immobilization throughout irradiation.

A single dose of 12 Gy whole-abdomen irradiation was delivered using a 6 MV linear accelerator device (Varian Medical Systems, Palo Alto, CA, USA) at a source-to-surface distance of 100 cm and a dose rate of 600 monitor units/min.

### 4.5. Rationale for Jejunum Selection

Jejunum tissue was selected because acute radiation enteropathy predominantly affects the rapidly renewing crypt–villus epithelium of the small intestine, where epithelial turnover, stem cell-dependent regeneration, and epithelial barrier maintenance are highly dynamic [8]. The jejunum is a well-established and biologically informative site for preclinical assessment of radiation-induced intestinal injury, and numerous experimental studies have used this intestinal segment to evaluate villus disruption, crypt degeneration, inflammatory injury, and epithelial barrier dysfunction following irradiation [3]. Restricting the analysis to a single predefined intestinal segment reduced the potential influence of region-dependent histomorphological variability and enabled internally consistent interpretation of histopathological alterations together with TNF-α, 5-LOX, Nrf2, and ZO-1 measurements.

### 4.6. Histopathological Evaluation of Small Intestine

#### 4.6.1. Tissue Processing and Histological Examination

Jejunum tissue samples obtained at the end of the experimental period were fixed in 10% neutral buffered formalin, routinely processed, and embedded in paraffin blocks. Paraffin-embedded tissues were sectioned at 4 μm thickness and stained with hematoxylin and eosin (H&E) for histopathological evaluation. All histological sections were examined and photographed using an Olympus BX51 light microscope equipped with an Olympus C-5050 digital camera (Olympus Corporation, Tokyo, Japan).

#### 4.6.2. Histopathological Scoring

Radiation-induced intestinal injury was evaluated semi-quantitatively using a study-specific modified histopathological scoring approach informed by previously described experimental radiation-induced intestinal injury criteria [58,59]. Histopathological alterations including epithelial disruption, villus injury, and Lieberkühn crypt degeneration were graded on a scale of 0–4 as follows:

0: Normal intestinal architecture with intact epithelium and villi, without inflammatory cell infiltration or hemorrhage.

1: Mild injury characterized by <25% epithelial and villus disruption together with focal Lieberkühn crypt degeneration.

2: Moderate injury with approximately 25% epithelial and villus disruption and evident Lieberkühn crypt degeneration.

3: Marked injury characterized by nearly 50% epithelial and villus disruption accompanied by widespread crypt degeneration.

4: Severe injury with >50% epithelial and villus destruction together with extensive Lieberkühn crypt degeneration.

All histopathological evaluations were performed under blinded conditions to minimize observational bias.

### 4.7. Biochemical Analysis of Small Intestine Tissue

#### 4.7.1. Tissue Homogenization and Protein Extraction

Frozen jejunum tissue samples were homogenized in phosphate-buffered saline (PBS, pH 7.2) containing protease inhibitors, including 1 mmol/L phenylmethylsulfonyl fluoride (PMSF), 1 mg/L pepstatin A, 1 mg/L aprotinin, and 1 mg/L leupeptin, using a glass homogenizer under cold conditions. The homogenates were subsequently centrifuged at 12,000 rpm for 20 min at 4 °C, and the resulting supernatants were collected for biochemical analyses. Total protein concentrations of the tissue supernatants were determined using the Bradford protein assay method [60].

#### 4.7.2. Measurement of TNF-α, 5-LOX, Nrf2, and ZO-1 Levels

The levels of tumor necrosis factor-alpha (TNF-α), 5-lipoxygenase (5-LOX), nuclear factor erythroid 2-related factor 2 (Nrf2), and zonula occludens-1 (ZO-1) in jejunum tissue homogenates were measured using commercially available enzyme-linked immunosorbent assay (ELISA, Bio-Techne (R&D Systems), Minneapolis, MN, USA) kits specific for rat samples, according to the manufacturers’ instructions. Total protein concentrations of the corresponding tissue homogenates were determined using the Bradford protein assay. For each sample, ELISA-derived analyte concentrations were normalized to total protein content. Results are expressed as pg/mg protein for TNF-α and ZO-1 and as ng/mg protein for 5-LOX and Nrf2.

### 4.8. Statistical Analysis

The distribution of data was evaluated using the Shapiro–Wilk normality test. Parametric data are expressed as mean ± standard deviation (SD). Comparisons among multiple groups were performed using one-way analysis of variance (ANOVA), followed by Tukey’s post hoc test for pairwise comparisons. Homogeneity of variances was assessed using the Brown–Forsythe test.

Histopathological injury scores did not show normal distribution; therefore, these data were expressed as median [interquartile range (IQR)] and analyzed using the Kruskal–Wallis test followed by Dunn’s multiple-comparison test for pairwise comparisons.

Statistical analyses were performed using GraphPad Prism software (version 9, GraphPad Software Inc., San Diego, CA, USA). A *p* value of <0.05 was considered statistically significant. For post hoc analyses, multiplicity-adjusted *p* values were used.

#### 4.8.1. Sample Size Determination

The sample size was estimated using G*Power version 3.1.9.7 based on effect estimates calculated from reported data in previous rat intestinal injury studies [61,62], which yielded Cohen’s f values of approximately 0.60–0.63 across relevant three-group comparisons. A conservative effect size of f = 0.60 was therefore used for the calculation. This assumption was also consistent with data from a comparable whole-abdomen radiation-induced intestinal injury model in rats [63]. With three groups, α = 0.05, and 80% power, the required total sample size was 30 animals (n = 10/group).

#### 4.8.2. Limitations

Several limitations should be acknowledged. Only female rats were included, precluding assessment of sex-dependent responses, and the analysis was restricted to the jejunum, limiting assessment of potential region-specific responses elsewhere in the intestine. The absence of a verapamil-only group limits distinction between radioprotective and intrinsic pharmacological effects of verapamil. Moreover, Nrf2 activation, 5-LOX activity, leukotriene production, oxidative stress, functional intestinal permeability, and tight-junction localization were not directly assessed; therefore, the biomarker changes should not be interpreted as evidence of functional pathway or barrier modulation. Macroscopic intestinal injury was not evaluated, and the use of single verapamil and radiation doses precluded dose–response assessment. Finally, the acute single-fraction model and short treatment period limit extrapolation to fractionated clinical radiotherapy, chronic radiation enteropathy, and prolonged verapamil exposure. The present study cannot characterize cumulative negative inotropic or conduction effects, long-term gastrointestinal tolerability, patient-selection criteria, monitoring requirements, or clinically consequential interactions with concomitant anticancer and supportive-care medications; these issues require dedicated evaluation before clinical translation [54,55,57].

## 5. Conclusions

The present study demonstrates that verapamil attenuates radiation-induced small-intestinal injury, as evidenced by reduced histopathological damage and TNF-α and 5-LOX levels, together with partial recovery of Nrf2 and ZO-1 levels. These findings support the potential of verapamil as a repurposing candidate for radiation-induced intestinal injury; however, the observed biomarker changes should be interpreted as associative rather than as evidence of functional pathway activation or preservation of intestinal barrier integrity. Further mechanistic and preclinical studies, followed by well-designed clinical trials, are required before verapamil can be considered for therapeutic application in patients with radiation-induced enteropathy.

## Data Availability

The data that support the findings of this study are not publicly available due to privacy/ethical reasons but are available from the corresponding author upon request.
